# Editorial: Genetics, evolution, and utilization of germplasm in crop improvement

**DOI:** 10.3389/fgene.2024.1527639

**Published:** 2024-12-16

**Authors:** Yang Zhu, Zhong-Hua Chen, Maximiller Dal-Biaco, Shuijin Hua

**Affiliations:** ^1^ Institute of Crop Science, Zhejiang University, Hangzhou, China; ^2^ School of Science, Western Sydney University, Penrith, NSW, Australia; ^3^ Departamento de Bioquímica, Universidade Federalde Viçosa, Viçosa, Minas Gerais, Brazil; ^4^ Institute of Crop and Nuclear Technology Utilization, Zhejiang Academy of Agricultural Sciences, Hangzhou, China

**Keywords:** genetics, evolution, germplasm, crop improvement, advanced technologies

Genetic diversity of different plant species is fundamental for trait development (Chung et al., 2023). By using either advanced sequencing or mutagenesis, knowledge of genetic diversity has been enriched in five newly published studies of *Prunus tenella*, *Lagerstroemia indica*, *Pisum sativum*, *Polygonati odorati* and *Ipomoea batatas*. Each study focused on specific genetic aspects, such as the *P. tenella* mitochondrial genome structure and unique gene transfer patterns, *L. indica* chloroplast genome emphasizing photosynthesis gene evolution and boundary shifts, and *P. sativum* SNP-based diversity highlighting population structure in landraces and cultivars. Together, these studies reveal phylogenetic relationships and adaptive traits, which could further support targeted breeding, conservation, and improved resilience in agricultural contexts. These findings collectively enrich genetic resources for many critical plant species.

Newly characterized levels and patterns of genetic diversity are critical for plant identification and feature specifications. There are three papers from different Chinese groups in this issue using genetic information from the cytoplasm, such as mitochondrial or chloroplast DNA evidence to distinguish plant genomic features. For example, in-depth analysis of the mitochondrial genome of the Chinese wild dwarf almond *P. tenella*, a rare and valuable plant with medicinal and ornamental potential, complements the chromosome level genome assembly (Qin et al., 2023). Using advanced Illumina and Oxford Nanopore sequencing platforms, the assembled mitochondrial genome of 452,158 bp in length contains 63 unique genes, comprising 36 protein-coding genes, 24 tRNA genes, and 3 rRNA genes (Liu et al.). However, differing from some other Prunus species, *P. tenella* exhibits unique repeat sequences, RNA editing sites, and intergenomic gene transfers between mitochondria and chloroplasts. Phylogenetic analysis places *P. tenella* closely with Rosaceae family members but highlights distinctions in its evolutionary pathway compared to *Prunus dulcis*, indicating divergent adaptation strategies within the genus.

By using the chloroplast genome structure of *Lagerstroemia indica* “Pink Velour” and six related species, intricate mechanisms of photosynthesis gene evolution have been revealed (He et al.). The high-resolution genome assembly contains 152,174 bp with a detailed annotation of 85 protein-coding genes, 37 tRNAs, and 8 rRNAs. Authors uncovered unique boundary variations in the *ycf1* gene across species, an evolutionary feature that distinguishes species like *Lagerstroemia fauriei* and *Lagerstroemia limii*. Another notable contribution is the use of nonsynonymous substitution rates (Ka/Ks) in photosynthesis genes, showing variation in *L. fauriei*, *L. limii*, and *Lagerstroemia subcostata* that indicate potential adaptive responses to differing climates (He et al.). Those findings are consistent with the previous study of divergence times of *Lagerstroemia* by using chloroplast phylogenomics of 35 species, which identified the *ycf1* gene as being quite variable during evolution (Dong et al., 2021).

For the four medicinal Polygonatum species, using codon usage bias (CUB) to analyze the codon preferences of 204 chloroplast protein-coding genes (PCGs) found the chloroplast genomes with weak codon usage bias (Shi et al.). These plant chloroplast genomes are enriched for AT bases and AT-ending codons. Natural selection is the main factor influencing codon usage bias, and mutation pressure also plays a role (Shi et al.). This study is of importance to distinguish *Polygonatum* plants, among which more than 30 species have been globally used as traditional medicine and functional food because of many chemical constituents with verified biological activities (Zhao et al., 2018).

Toward improvement of agronomic traits, a population with natural genetic variation or generated mutagenesis pools is of great importance for breeding or pre-breeding programs (Holme et al., 2019). This Swedish group used 265 globally sourced accessions of pea (*P. sativum)*, applying advanced Diversity Arrays Technology (DArT) sequencing to identify 6,966 SNP and 8,454 *in silico* markers (Brhane and Hammenhag). This highly informative genetic dataset exhibited the highest diversity (Ne = 1.52, He = 0.31), with unique private alleles primarily in European accessions (22 alleles), making these groups particularly valuable for future breeding (Brhane and Hammenhag). Notably, the reference genome of the elite vegetable pea cultivar “Zhewan No.1” has been recently released, providing genetic information relevant to many agronomic traits (Liu et al., 2024).

A Korean group chose to apply gamma radiation to the sweetpotato (*I. batatas*) cultivar “Tongchaeru” for genetic mutagenesis. With the aim of altered stem growth patterns, authors combined transcriptomic changes and genetic alterations for agronomic trait changes (Lee et al.). In summary, researchers identified notable phenotypic changes in stem morphology, such as longer or thinner stems in mutants compared to the wild type. Transcriptomic analysis detected 15,832 differentially expressed genes, with critical upregulation in the auxin-response gene *SAUR* and *PIF4*, a key gene for cell elongation (Lee et al.). This suggests that gamma-induced mutations can enhance auxin and gibberellin pathways, promoting stem elongation. Due to the highly heterozygous hexaploid genome of *I. batatas*, complicated genetic studies and breeding programs have been comprehensively reviewed for better sweetpotato improvement (Yan et al., 2022).
